# 

**Published:** 1884-01

**Authors:** 


### THE BISTOURY:

Thad S. Up de Graff, M. D., Editor & Proprietor.

### *CONTENTS FOR JANUARY, 1884.*

**CONTRIBUTIONS.** PAGE.

How Do Men Become Doctors? ........ Ben. H. Pratt ........ 287

Names ........ Ausburn Towner ........ 290

Infinitessimalism Again ........ C. S. Carr, M. D ........ 292

What Of It? ........ Geo. E. Blackham, M. D ........ 294

Culture ........ J. B. Chandler ........ 296

Invisible Blood Corpuscles ........ J. M. Adams ........ 298

**MEDICAL ITEMS AND NEWS.**

Typhoid and Intoxication—Gastric Ulcer—Gastralgia—Tonsilitis—Malarial Hematuria—Syphilis and Vaccinia, Antagonism Between—Cancer—Erysipelas—Spasmodic Cough—For Imperfection in Speech—Winter Eczema—Zoster, Oil of Peppermint in—Tin Poisoning—Condylomata—Infectious Diseases—Kissing, Danger of—Chalky Deposits—Syphilis—Weak Heart, Electricity vs. Chloroform—Albuminuria—Trichinosis Nodules—Scars—Spasms of the Œsophagus—Whooping Cough—Secondary Syphilis—Hæmoptysis—Mercurial Poisoning—Lumbricoides—Ear, Abscess of the Lobule of the—Dyspnœa—Angina Faucium—Primary Cancer of the Lung—Fetid Bronchitis—Gall Bladder, Excision of the—Sewer Gas Poisoning—Nocturnal Incontinence of Children—Typhus and Typhoid—Mixed Chloroform ........ 299 to 302

**HOUSEHOLD HINTS AND RECIPES.**

A Bronze—A Disinfectant Laundry Blue—Label Varnish—Restoring the Finish of Leather—To Keep Wooden Posts from Rot—Thomas's Electric Oil—Imitation Walnut—Preservation of Fruit—Grape Leaves for Pickles—Black Lacquer for Coating Bottles—To Clean Brass—Shaving Soap—Nickel Plating—To Embalm Small Quadrupeds—Prize Receipt for Salad ........ 303-304

**EDITORIAL DEPARTMENT.**

A Small Subscription—The Index—" A Merry Christmas"—Danger in Inhumation—" Before The Class." . 305-306

**TERMS.—50 Cents a Year. Address the Bistoury, Elmira, N. Y.**

